# Posterior Reversible Encephalopathy Syndrome in Clinical Toxicology: A Systematic Review of Published Case Reports

**DOI:** 10.3389/fneur.2019.01420

**Published:** 2020-02-12

**Authors:** Bérenger Largeau, David Boels, Caroline Victorri-Vigneau, Clara Cohen, Charlotte Salmon Gandonnière, Stephan Ehrmann

**Affiliations:** ^1^CHU de Nantes, Service de Pharmacologie Clinique—Centre Régional de Pharmacovigilance, Nantes, France; ^2^CHU de Nantes, Service de Pharmacologie Clinique—Unité de Toxicologie Clinique et Toxicosurveillance Médicamenteuse, Nantes, France; ^3^Université de Nantes, Université de Tours, INSERM, Methods in Patients-Centered Outcomes and Health Research (SPHERE)—UMR 1246, CHU de Nantes, Service de Pharmacologie Clinique—Centre d'Évaluation et d'Information sur la Pharmacodépendance et d'Addictovigilance, Nantes, France; ^4^Université de Tours, CHRU de Tours, Service de Neuroradiologie Diagnostique et Interventionnelle, Tours, France; ^5^CHRU de Tours, Service de Médecine Intensive Réanimation, CIC 1415, réseau CRICS-TRIGGERSEP, Tours, France; ^6^Université de Tours, INSERM, Centre d'Étude des Pathologies Respiratoires (CEPR)—UMR 1100, CHRU de Tours, Service de Médecine Intensive Réanimation, CIC 1415, Réseau CRICS-TRIGGERSEP, Tours, France

**Keywords:** leukoencephalopathy syndrome, hypertensive encephalopathy, blood–brain barrier, substance abuse, alcohol, poisoning

## Abstract

**Background:** Posterior reversible encephalopathy syndrome (PRES) is a rare clinical and radiological entity characterized by a typical brain edema. Although several case reports have described PRES in a context of poisoning, to our knowledge, a comprehensive assessment has not been performed. The aim of this systematic review was to raise awareness on poisoning-specific PRES features and to encourage consistent and detailed reporting of substance abuse–and drug overdose–associated PRES.

**Methods:** Medline/PubMed, Web of Science, and PsycINFO were screened through May 31, 2019, to systematically identify case reports and case series describing PRES associated with poisoning (i.e., alcohol, drugs, illicit drugs, natural toxins, chemical substances) in accidental context, intentional overdose, and substance abuse. The methodological quality of eligible case reports/series was assessed. Patients and exposure characteristics were recorded; relevant toxicological, radiological, and clinical data were extracted.

**Results:** Forty-one case reports and one case series reporting 42 unique cases were included. The median time to PRES onset from the start of exposure was 3 days (IQR 2–10). Acute high blood pressure, visual disturbance, and seizure were reported in 70, 55, and 50% of patients, respectively. The initial clinical presentation was alertness disorders in 64% of patients. Nine patients (21%) required mechanical ventilation. One-third of patients had at least one risk factor for PRES such as chronic hypertension (17%) or acute/chronic kidney failure (24%). The main imaging pattern (67%) was the combination of classical parieto-occipital edema with another anatomical region (e.g., frontal, basal ganglia, posterior fossa involvement). Vasogenic edema was found in 86% of patients. Intracranial hemorrhage occurred in 14% of patients. Both brain infarction and reversible cerebral vasoconstriction syndrome were diagnosed in 5% of patients. Three patients (12%, 3/25) had non-reversible lesions on follow-up magnetic resonance imaging. The median time required to hospital discharge was 14 days (IQR 7–18). Mortality and neurological recurrence rate were null.

**Conclusions:** Comorbidities such as chronic hypertension and kidney failure were less frequent than in patients with other PRES etiologies. Imaging analysis did not highlight a specific pattern for poisoning-induced PRES. Although less described, PRES in the context of poisoning, which shares most of the clinical and radiological characteristics of other etiologies, is not to be ignored.

## Introduction

Posterior reversible encephalopathy syndrome (PRES) is a rare radiological and clinical entity characterized by a typical brain edema and various symptoms such as high blood pressure (75–80%), encephalopathy (50–80%), headache (50%), visual disturbances (33%), focal neurological deficits (10–15%), seizures (60–75%), and status epilepticus (5–15%) (1). Kidney injury is highly prevalent during PRES (up to 55%), and more than half of patients have chronic hypertension (1). PRES can occur in a number of complex clinical conditions, classically dichotomized into iatrogenic (e.g., antineoplastic therapy, calcineurin inhibitors) and PRES-associated medical conditions (e.g., eclampsia, sepsis, autoimmune disorders), requiring mechanical ventilation for 3–7 days in 35–40% of patients (2). Although there is currently no unified diagnostic algorithm, neuroimaging usually yields bilateral cortical–subcortical vasogenic edema according to three anatomic patterns: dominant parieto-occipital involvement, variant atypical PRES, and combination of different patterns. Variant atypical PRES gathers superior frontal sulcus, holohemispheric watershed, cerebellum, basal ganglia, brainstem, and spinal cord involvements (3, 4). The atypical or combined patterns are more common than the typical PRES with isolated parieto-occipital involvement.

The pathophysiology of PRES is still debated through hypoperfusion and hyperperfusion theories. Impaired cerebral autoregulation responsible for disruption of the blood–brain barrier (BBB) is one of the two main hypotheses, the other one being endothelial dysfunction (5). A recent review suggests that arginine vasopressin (AVP) axis stimulation could precipitate PRES development through an increase in AVP secretion or AVP receptor density. Activation of vasopressin V1a receptors leads to cerebral vasoconstriction, endothelial dysfunction, and subsequent brain edema (5).

Thus, PRES is a syndrome whose circumstances of occurrence and radiological features are associated with significant clinical and radiological polymorphism, making it difficult to characterize. Drug toxicity is one of the potential etiologies. Data are available in the literature regarding PRES occurring at therapeutic drug doses, but to the best of our knowledge, no review focused specifically on cases of accidental or intentional poisoning-associated PRES.

In order to investigate the occurrence of PRES in poisoned patients, the authors systematically reviewed the scientific literature of case reports and case series concerning PRES associated with poisoning (i.e., alcohol, drugs, illicit drugs, natural toxins, chemical substances) in accidental context, intentional overdose, and substance abuse. The authors did not include cases of calcineurin inhibitor overexposure, which has already been covered (6). The purpose of this study is to raise awareness of the features of PRES in poisoned patients and encourage consistent and detailed reporting of PRES in a context of overdose and substance abuse.

## Methods

A systematic review was conducted in accordance with the Preferred Reporting Items for Systematic reviews and Meta-Analyses (PRISMA) guidelines (7).

### Eligibility Criteria

All language case reports and cases series were eligible if they concerned human subjects and included full-text. Records were screened if they were explicitly labeled as PRES based on head MRI (magnetic resonance imaging) or CT (computed tomography) scans. Case reports were included if PRES occurred in temporal connection with poisoning (i.e., alcohol, drugs, illicit drugs, chemical substances, natural toxins) in accidental context, intentional overdose, or substance abuse and/or a causal relationship was assumed by the authors of the report.

### Data Sources, Search Strategy, and Data Extraction

A search using Medline/PubMed, Web of Science and PsycINFO was performed without any limits through May 31, 2019. The search terms in Medline were a combination of medical subject heading (MeSH) terms and keywords. The search strategy consisted of using the health multi-terminology portal HeTOP (8) for each term (i.e., text words OR MeSH OR title/abstract) with the following search algorithm: ([posterior leukoencephalopathy syndrome] AND ([poisoning] OR [overdose] OR [intoxication] OR [substance abuse] OR [solvents] OR [organophosphates] OR [licorice] OR [venoms] OR [scorpion] OR [mushroom] OR [lysergic acid] OR [cathinone] OR [cocaine] OR [amphetamine] OR [heroin] OR [cannabis] OR [alcohol])). A manual search and screening of the bibliographies of selected articles was performed, in addition to the computerized search. The search strategy is summarized in Supplementary Figure 1.

Extracted data included the following clinical and toxicological considerations: age, gender, risk factors of PRES occurrence, and exposure characteristics to the causative agent. Clinical and radiological data on PRES, its time course, and outcomes were also extracted. Unreported data were considered absent. Neuroimaging results were classified according to three different patterns. Briefly, the classical PRES pattern, involving only parieto-occipital lobes; the variant, including isolated various anatomical regions (e.g., frontal, cerebellum, brainstem, basal ganglia); and finally, the combined pattern, which includes combination of various regions.

### Methodological Quality Assessment

The methodological quality of case reports and cases series was assessed using the tool proposed by Murad et al. (9) modified to adapt it to the analysis of toxicological exposures associated with PRES. The selected items were based on scores used for causality assessment in drug overdose (10, 11). This new tool converges into 10 items that can be categorized into six domains: selection, ascertainment, diagnosis, causality, follow-up, and reporting (Supplementary Table 1). Briefly, items included time to onset of PRES, exposure characteristics (e.g., dose, drug detection, identification of species), radiological features, clinical data (e.g., symptoms, risk factors), dechallenge phenomenon, differential diagnosis, pharmacological properties of the causative agent, and clinical/radiological follow-up. The results of this modified tool have been summarized by summing the scores of the 10 binary responses into an aggregate score (Supplementary Tables 1, 2).

## Results

### Case Selection

The literature search revealed a total of 149 non-duplicate records of which 95 were excluded because they did not report poisoning-associated PRES or were not case reports or case series. Out of 54 full-texts assessed for eligibility, 13 were excluded because they did not report poisoning-associated PRES or outcomes of interest (i.e., symptoms, brain edema features, follow-up). Finally, 40 case reports and 1 case series reporting a total of 42 unique cases were included (Supplementary Figure 1).

### Synthesized Findings

#### Demographic Characteristics and Clinico-Biological Findings

Demographic data, substances involved, and clinico-radiological characteristics are summarized in Supplementary Table 3. Out of 42 patients included, 22 were female (52%); median age was 41 years (IQR 27–55, range 3–73). Four children were included (12–15). The median time to PRES onset from the start of exposure or intoxication diagnosis was 3 days (IQR 2–10) and ranged from 2 h (13) to 4 months (16, 17). The initial clinical presentation was alertness disorders in 64% of patients (13–15, 18–40). Visual disturbances were reported in 55% of patients (23/42) (12, 13, 15, 16, 19–21, 23, 25–27, 29, 34, 35, 37, 39, 41–47) including photophobia (19, 20, 35), visual hallucination (21, 23, 26, 27), and cortical blindness (15, 16, 24, 29, 34, 37). Four patients had concurrent acute kidney failure with PRES (13, 19, 44, 48). When performed (33, 39, 43), hypomagnesemia was found in one patient (39). Cerebral spinal fluid (CSF) analysis (20, 21, 23, 24, 34, 36, 37, 45, 47, 49) showed elevated CSF protein in one patient (34). Nine patients (21%) required mechanical ventilation (14, 21, 28, 33, 38, 46–48). The median time required to hospital discharge was 14 days (IQR 7–18). Mortality and neurological recurrence rate were null.

#### Radiological Features

Radiological diagnosis was based on brain MRI and CT scans in 91% (38/42) and 9% (4/42) (35, 38, 40, 43), respectively. The neuroimaging findings, including anatomical pattern, diffusion-weighted imaging (DWI), apparent diffusion coefficient (ADC), edema type, symmetry, and arterial characteristics, are summarized in Supplementary Table 3. Five patients (19, 24, 26, 34, 44) had normal initial brain CT (5/9, 56%) (19, 20, 24, 26, 34, 42, 44, 48, 50). All patients exhibited cortical and/or subcortical edema, characterized by hyperintensities in T2-weighted and/or T2 fluid-attenuated inversion recovery (FLAIR) imaging, which was consistent with PRES diagnosis in MRI. Symmetrical lesions were reported in 76% of patients (25/33) (12–15, 19, 22–25, 27, 29, 32–34, 36, 37, 39, 40, 43, 44, 46, 47, 49, 50). The combined pattern, which includes combination of various regions, was the most frequent with 67% of patients (13, 14, 17–22, 24–26, 28, 30–35, 38, 39, 41, 44, 46–49, 51). The classical PRES pattern, involving only parieto-occipital lobes, was found in 19% (8/42) of patients (12, 16, 23, 27, 37, 40, 42, 45). The variant pattern of PRES (6/42, 14%), including isolated anatomical region such as occipital (29, 36, 43, 50) and cerebellum (15, 52), occurred in 9 and 5%, respectively. In five reports, angiography (by contrast-enhanced arteriography in CT, or MRI sequences including non-contrast time-of-flight and angio-MRI or digital subtraction angiography) showed narrowing of the cerebral arteries (16, 28, 36, 37, 49). Reversible cerebral vasoconstriction syndrome (16, 49) was diagnosed in 5% of patients (2/42).

DWI was positive in 60% of patients (12/20), totally or partially in correspondence with T2/FLAIR hyperintensities, with focal or larger extent (20, 25, 26, 29, 30, 36, 37, 39, 47–49). When data related to ADC were provided, positive DWI due to vasogenic edema (i.e., no restricted ADC) (16, 17, 19, 21, 23, 25, 26, 28–31, 33, 34, 36, 45, 47–49) and cytotoxic edema (i.e., restricted ADC) (20, 24, 39, 49) were found in 86 and 19% of cases, respectively. T2^*^ (gradient echo) or susceptibility weighted imaging revealed intracranial hemorrhage in 14% of patients (16, 20, 30, 31, 36, 43). The median time of follow-up imaging was 21 days (IQR 11–60). Out of 25 patients with follow-up imaging, 22 (88%) showed complete resolution of brain edema (12–14, 16, 21, 22, 24, 26, 29–31, 33, 38, 43–47, 50, 51).

### Characteristics According to the Causative Agent

Exposure characteristics, clinico-radiological features, and follow-up data of the included cases are shown in Table 1.

**Table 1:** Characteristics of the 42 included cases.

**References**	**Substance/context**	**Sex/age**	**Comorbidities**	**Time to onset**	**Blood pressure**	**Diagnosis**		**Follow-up**	
						**Symptoms**	**Imaging**	**Outcomes**	**Imaging**
Bhagavati et al. (22) #2	Acute alcohol intoxication	M/57	Chronic alcohol abuse	2 days	131/88	Confusion	Combined and symmetrical patterns	Complete clinical resolution 2 weeks after PRES	Complete resolution after 2 weeks
Coppens et al. (23)	Acute alcohol intoxication	M/43	Chronic alcohol abuse on disulfiram	3 weeks after daily alcohol consumption	150/100	Disorientation, delirium, visual hallucination, paraparesis	Parieto-occipital and symmetrical patterns, normal diffusion on DWI	Slight improvement of lower limb paresis over several months	Almost complete resolution at day 14
Srikrishna et al. (24)	Acute alcohol intoxication	M/68	Chronic alcohol abuse on disulfiram	3 h after alcohol consumption	160/100	Vomiting, headache, confusion, cortical blindness, seizure	Combined and symmetrical patterns, positive DWI, restricted diffusion on DWI	Clinical improvement after 15 days Walk without any assistance after 21 days	Complete resolution at day 21
Kim et al. (25)	Acute alcohol intoxication	M/59	Chronic alcohol abuse	1 day	191/100	Visual disturbance, aphasia	Combined and symmetrical patterns, positive DWI	Discharge at day 14	Only slight resolution after 2 months
Ishikawa et al. (26)	Alcohol withdrawal and acute pancreatitis	F/28	Ø	2 days	112/82	Confusion, disorientation, visual hallucination, seizure	Combined pattern, positive DWI, no restricted ADC	General condition improved at day 7 Discharge at day 17	Complete resolution at day 16
Mengi et al. (27)	Alcohol withdrawal	M/53	Ø	3 days	150/90	Disorientation, visual hallucination	Parieto-occipital and symmetrical patterns	Clinical resolution at day 7	Significant regression at day 7
Gill et al. (28)	Alcohol withdrawal after admission for acute alcohol intoxication and AKI	F/58	Chronic HTN	2 days	195/108	Coma, obtundation	Combined pattern, no restricted ADC Subacute infarct in the right parietal lobe	Discharge at day 2	Resolution (date unknown)
Kimura et al. (29)	Chronic alcohol abuse	M/51	Amphetamine abuse	Not applicable	170/100	Confusion, disorientation, cortical blindness	Atypical and symmetrical patterns, positive DWI	Clinical symptoms resolved in 4 days No permanent neurological abnormalities after 1 month	Complete resolution after 1 month
Baek et al. (30)	Chronic alcohol abuse with acute pancreatitis	M/49	Ø	Concomitantly with acute pancreatitis	130/80	Confusion, disorientation	Combined pattern, positive DWI Microbleeds in cerebellum	Mental status improved after 2 days Able to walk around at day 5 Discharge at day 14	Complete resolution, except few microbleeds in cerebellum after 1 month
Magno Pereira et al. (31)	Chronic alcohol abuse	M/33	Ø	Not applicable	190/100	Headache, vomiting, confusion, seizure	Combined pattern, no restricted diffusion Subacute hemorrhage in the right frontal lobe	Discharged at day 15	Signs of hemorrhage reabsorption after 2 months
Murphy et al. (41)	Chronic alcohol abuse with acute pancreatitis	F/40	Ø	1 week after acute pancreatitis	Not available	Visual loss	Combined pattern	Visual recovery after 2 months	Lesions improved after 2 months
John et al. (32)	Severe acute alcoholic hepatitis	F/40	Hepatic encephalopathy	3 weeks after alcoholic hepatitis diagnosis	114/78	Headache, psychomotor retardation, seizure	Combined and symmetrical patterns	Not available	Not available
Fitzgerald et al. (33) #1	Lithium withdrawal after lithium intoxication with AKI	F/50	Chronic HTN	9 days after intoxication	140/80	Headache, bladder/bowel incontinence, seizure	Combined and symmetrical patterns, no restricted diffusion	Discharged at day 22	Complete resolution 5 months later
Fitzgerald et al. (33) - #2	Lithium withdrawal after lithium intoxication with AKI	M/61	Chronic HTN	8 days after intoxication	SBP = 170	Mental status altered	Combined and symmetrical patterns	Discharged at day 25	Not available
Loens et al. (34)	Lithium withdrawal after lithium intoxication with AKI	F/60	Chronic HTN	3 days after intoxication	MAP > 130	Somnolence, disorientation, dysarthria, cortical blindness, seizure	Combined and symmetrical patterns, no restricted diffusion on ADC	ICU for 10 days Full recovery after 22 days	Regression of the edema with residual bi-occipital lesions at day 10
Minhaj et al. (35)	Dextroamphetamine and clonidine overdose	M/17	Ø	9 h	182/111	Headache, photophobia, confusion	Combined pattern	Asymptomatic at 36 h and had no neurologic sequelae	Not available
Mann et al. (51)	Acetaminophen overdose with AKI at day 3	F/21	Ø	10 days	164/103	Seizure	Combined pattern	Discharged at day 15 Asymptomatic at day 20	Complete resolution at day 20
Kinno et al. (49)	Ephedrine overdose	M/28	Ø	3 days	Not available	Paraplegia, agraphia	Combined and symmetrical patterns, positive DWI, co-existence of restricted and no restricted ADC Brain infarction with RCVS and PRES	Not available	Residual lesions in the parietal lobe at day 27
Kawanabe et al. (36)	Phenylpropanolamine misused as eye drops for 4 days	F/57	Ø	3 days after the last dose	186/106	Headache, somnolence, seizure	Atypical and symmetrical patterns, positive DWI, no restricted ADC Intraparenchymal left occipital hemorrhage	Discharge at day 34	Nearly complete resolution after 2 months
Weidauer et al. (37) #1	Digitoxin overdose	F/73	Not available	3 days	Normal	Disorientation, vomiting, cortical blindness	Parieto-occipital and symmetrical patterns, positive DWI	Slowly improvement of visual acuity over 4 months	Not available
Akinci et al. (15)	Bismuth overdose with AKI at day 3 requiring HD	F/16	Ø	15 days	110/70	Confusion, somnolence, cortical blindness, ataxia, seizure	Atypical and symmetrical patterns	Discharged 9 days after PRES	Not available
Bazuaye-Ekwuyasi et al. (18)	Cocaine	F/41	HIV Chronic HTN ESRD	Not available	198/92	Confusion, agitation	Combined pattern	Confusion and agitation slowly resolved at day 5	Complete resolution at day 7
Dasari et al. (19)	Cocaine	F/40	Chronic HTN AKI on CKD	4 days after heavy cocaine binging	189/140	Headache, somnolence, vomiting, photophobia	Combined and symmetrical patterns, no restricted diffusion on DWI	Discharged after 2 days	Not available
Tantikittichaikul et al. (52)	Amphetamine	M/45	Not available	Not available	SBP = 250	Headache	Atypical variant and no restricted	Not available	Significant improvement of the lesions at day 3
Omer et al. (50)	Mephedrone	F/19	Alcohol abuse	2 days	SBP > 160	Seizure	Atypical and symmetrical patterns	No recurrence of seizure at day 10	Complete resolution at day 10
Castillo et al. (20)	Kratom	M/22	Amphetamine, marijuana abuse	“Prior to admission”	180/105	Headache, disorientation, photophobia, aphasia	Combined pattern, positive DWI, restricted diffusion Left parieto-occipital intraparenchymal bleed	Discharged after 5 days	Lesions unchanged at day 4
Legriel et al. (21)	Lysergic acid amide	M/39	Alcohol abuse	“Immediately”	185/130	Confusion, hyperreflexia, visual hallucination, seizure	Combined pattern	Discharged after 9 days in the ICU Normotensive 1 month later	Complete resolution at day 7
Delgado et al. (38)	Snake bite (*Bothrops asper*)	M/18	Ø	2 days	<140/90	Headache, respiratory distress, stuporous, seizure	Combined pattern	Discharged asymptomatic 1 month later	Completely normal at 6 months
Varalaxmi et al. (42)	Pit viper bite with AKI requiring HD at day 3	M/45	Ø	6 days	140/90	Headache, vision loss	Parieto-occipital pattern	Normal vision within 24 h	Not available
Ibrahim et al. (39)	Horned viper bite (*Cerastes cerastes*)	F/23	Ø	“Within a week”	130/80	Stuporous, vision loss	Combined pattern, positive DWI, restricted ADC	Normotensive within 1 week Persistence of vision loss 3 months later	Not available
Kaushik et al. (12)	Indian krait bite (*Bungarus caeruleus*)	M/10	Ø	15 days	“HTN”	Visual loss, seizure	Parieto-occipital and symmetrical patterns	Normal vision within 2 days Normotensive 1 month later	Complete resolution after 3 months
Marrone et al. (13)	Scorpion sting (*Tityus bahiensis*)	M/13	Ø	2 h	90/60	Vomiting, headache, visual disturbance, obnubilation, seizure	Combined and symmetrical patterns	Asymptomatic at day 5	Complete resolution at day 21
Rebahi et al. (14)	Scorpion sting (*Androctonus mauretanicus*)	F/3	Ø	2 days	170/110, then cardiogenic shock	Vomiting, impaired consciousness, seizure	Combined and symmetrical patterns	Discharge at day 18 with monoparesis and low visual acuity	Complete resolution at 6 months
Loh et al. (40)	Wasp stings complicated by AKI requiring HD	F/29	Ø	1 month	190/104	Loss of consciousness	Parieto-occipital and symmetrical patterns	Blood pressure and symptoms resolved spontaneously on the same afternoon	Not available
Du et al. (48)	Wasp stings	F/66	Not available	1 day	135/85	Headache, seizure	Combined pattern, positive DWI	Not available	Not available
Chatterjee et al. (16)	Licorice	F/49	Ø	4 months	230/130	Headache, cortical blindness, hyperreflexia, seizure	Parieto-occipital pattern Bilateral lobar hemorrhage RCVS with segmental narrowing of multiple intracranial arteries	Headache resolved at day 8 Asymptomatic at 1 month	Resolution of PRES, RCVS, and lobar hemorrhage after 3 months
Van Beers et al. (43)	Licorice	F/49	Ø	2 weeks	219/100	Headache, visual impairment	Atypical and symmetrical patterns Microbleeds in the left sylvian fissure	Quickly recovery and discharged after few days	Complete resolution at day 10
Morgan et al. (44)	Licorice	F/65	Ø	4 days after consumption	SBP > 220	Confusion, headache, loss of vision	Combined and symmetrical patterns	Discharged at day 9	Complete resolution after 2 months
O'Connell et al. (45)	Licorice	F/56	Chronic HTN	“Since recent months”	210/80	Headache, nausea, visual disturbance, seizure	Parieto-occipital pattern, no restricted diffusion	Not available	Complete resolution at day 21
Tassinari et al. (17)	Licorice	M/10	Ø	4 months	144/102	Headache, seizure	Combined pattern	Normotensive 1 month later	Significant regression at day 14
Zhou et al. (46)	Mushroom (*Unidentified*)	F/26	Ø	12 h	190/130	Seizure, coma	Combined and symmetrical patterns	Fully recovered within 2 days	Complete resolution (date unknown)
Phatake et al. (47)	Organophosphate	M/32	Ø	4 days	200/110	Headache, visual disturbance, seizure	Combined and symmetrical patterns, positive DWI, no restricted ADC	Improvement in vision and mental state 8 days after PRES Discharge at day 17	Complete resolution at day 24

Ø*, no comorbidity related to PRES; ADC, apparent diffusion coefficient; AKI, acute kidney injury; CKD, chronic kidney disease; DBP, diastolic blood pressure; DWI, diffusion-weighted imaging; ESRD, end-stage renal disease; HTN, hypertension; HD, hemodialysis; HIV, human immunodeficiency virus; ICU, intensive care unit; MAP, mean arterial pressure; RCVS, reversible cerebral vasoconstriction syndrome; SBP, systolic blood pressure*.

#### Alcohol

In alcohol-induced PRES, three different situations have been described: chronic alcohol intoxication (29–32, 41), acute alcohol intoxication (22–25), and alcohol withdrawal (26–28). PRES in chronic alcohol abusers occurred in conjunction with other complications of alcoholism such as acute pancreatitis (26, 30, 41) and hepatic encephalopathy due to severe acute alcoholic hepatitis (32). PRES onset occurred at the same time as acute pancreatitis (26, 30) or 1 week later (41), whereas it occurred 3 weeks after acute alcoholic hepatitis onset (32). All but four patients were hypertensive. Patients were normotensive in acute alcoholic pancreatitis (26, 30) and acute alcoholic hepatitis (32). Headache was reported in only 17% of patients (2/12) (24, 32). In three patients, PRES was complicated by intracranial hemorrhage (30, 31) or brain infarction (28).

#### Drug Overdose

Nine patients experienced PRES in a context of drug overdose, involving lithium (33, 34), dextroamphetamine (35), acetaminophen (51), ephedrine (49), phenylpropanolamine (36), digitoxin (37), and bismuth (15). The median time to PRES onset from the intoxication was 3 days, and two distinct situations were identified. PRES occurred during the acute phase of poisoning (35–37, 49) or at distance from intoxication (i.e., after stopping the causative drug) (15, 33, 34, 51). Three case reports indicated an association between lithium and PRES (33, 34). All these cases occurred after lithium discontinuation in patients with hypothyroidism, with chronic hypertension, and for whom intoxication was complicated by acute kidney injury (33, 34). Acute kidney injury occurred before the onset of neurological disturbances in five patients (56%) (15, 33, 34, 51). Angiography showed narrowing of the cerebral arteries in three cases (36, 37, 49). Infarction (49) and intraparenchymal hemorrhage (36) were also reported.

#### Illicit Drug

We collected six reports of illicit drug–induced PRES, involving cocaine (18, 19), amphetamine (52), mephedrone (50), kratom (20), and lysergic acid amide (21). Acute high blood pressure was reported in all patients (18–21, 50, 52). Patients had several risk factors for PRES such as chronic hypertension (18, 19), chronic kidney disease (18, 19), alcohol abuse (21, 50), and HIV infection (18).

#### Natural Toxin

In venom-induced PRES, snake bites [*Cerastes cerastes* (39), *Bothrops asper* (38), pit viper (42), *Bungarus caeruleus* (12)] were involved in four patients, scorpion stings in two cases [*Tityus bahiensis* (13), *Androctonus mauretanicus* (14)], and multiple wasp stings in two patients (40, 48). PRES was associated with mushroom (46) and licorice (16, 17, 43–45) in one and five patients, respectively. In 36% (5/14) of patients (12, 16, 17, 40, 43), the time from exposure start to PRES onset was more than 1 week. Patients required mechanical ventilation in 29% (4/14) of cases (14, 38, 46, 48); the duration of mechanical ventilation ranged from 2 (46) to 14 days (38). Acute kidney failure occurred in 36% (5/14) of patients (13, 40, 42, 44, 48), requiring hemodialysis in two cases (40, 42). Among the five patients with licorice-associated PRES, brain hemorrhage occurred in two patients (16, 43), one of which was associated with reversible cerebral vasoconstriction syndrome (16).

#### Chemical Substance

Phatake et al. (47) reported a patient complaining of headache and blurred vision 4 days after coma induced by consumption of organophosphorus compounds with suicidal intention. Brain MRI showed multifocal hyperintensities mainly in subcortical areas of parietal and occipital areas with increase ADC, suggesting PRES.

## Discussion

### Are Toxic PRES Different From Other Etiologies?

As in other etiologies, poisoning-associated PRES are very polymorphic in terms of both exposure characteristics and patient comorbidities. Regardless of the substances involved, the median of 3 days for time to onset of PRES can dichotomize the presentation of this syndrome into two distinct situations. Indeed, PRES occurring after 3 days seems to be more related to a rebound effect of intoxication or to complications related to the management of the poisoning, whereas a shorter period would support a direct link between the causative agent and PRES.

#### Clinical and Biological Findings

Clinically, the prevalence of symptoms usually reported in PRES patients was consistent with the published literature (1). Indeed, acute high blood pressure, visual disturbance, and seizure were reported in 70, 55, and 50% of patients, respectively. Seizure is the most common symptom found in children (53), and all children had seizures (12–15). However, comorbidities such as chronic hypertension (17%, 7/42) and acute or chronic kidney failure (24%, 10/42) were less frequently reported than in patients with other PRES etiologies, where their estimated prevalence is about 50% (1). In the literature, the frequency of isolated CSF protein elevation without pleocytosis, as a biomarker of permeability disruption of the BBB, varies from 60% (54) to 75% (55). In this review, although few cases reported CSF analysis, only one case (1/10, 10%) (34) described elevated CSF protein.

#### Radiological Features

All reviewed patients presented cortical and/or subcortical T2/FLAIR hyperintensities or hypodensities on CT when MRI was not performed, which was consistent with the main findings of PRES (3, 4). These hyperintensities correspond to the brain edema caused by vascular dysregulation, leading to acute vasodilatation and classically vasogenic edema, with proven pathological/imaging correlation (56). While parieto-occipital involvement (12–14, 16–18, 20–34, 36–51) was predominant (39/42, 93%), as described in the literature (57), the isolated parieto-occipital was not the main pattern in this review, supplanted by the pattern combining various anatomical regions involved. Frontal (13, 17, 18, 20, 22, 24–26, 28, 31, 33, 38, 44, 46, 48, 49, 51), temporal lobe (18, 25, 26, 30, 32, 33, 47), and cerebellum (14, 15, 18–21, 30, 35, 41, 52) involvement occurred in 41% (17/42), 17% (7/42), and 24% of patients (10/42), respectively. In this review, the prevalence of frontal and temporal involvement is lower than previously described, where it can be seen in up to 75% of cases (3). Indeed, temporal involvement was rarely reported, even though MRI images seemed to show involvement in this region. This may partially explain the difference in prevalence, especially since the whole brain MRI was not available for neuroradiological analysis.

The atypical distribution involving the thalamus (18, 19, 21, 34), basal ganglia (18, 19, 46), midbrain (18, 19), and corpus callosum (39) was less commonly reported, as described in the literature. As with other PRES etiologies, atypical imaging appearances including hemorrhage, contrast enhancement, and restricted diffusion on MRI were reported in similar proportions (58). Intracranial hemorrhage occurred in 14% of cases included (6/42) (16, 20, 30, 31, 36, 43). Among these cases, minimal occipital intraparenchymal (20, 36), microbleeds in the cerebellum (30) and sylvian fissure (43), and subarachnoid hemorrhage (31) were reported. Regarding contrast enhancement (19, 20, 22, 23, 30, 31, 38, 52), only one case described gyriform enhancement (20). In this case of PRES induced by kratom, brain MRI showed multifocal areas of abnormal T2 FLAIR; restricted diffusion in the superior parietal lobes, both occipital lobes, and both cerebellar hemispheres; and minimal hemorrhage in the left superior parietal lobe consistent with atypical PRES (20). DWI positivity (20, 25, 26, 29, 30, 36, 37, 39, 47–49) due to advanced edema was higher (12/20, 60%) than previously described in other PRES etiologies. The occurrence of cytotoxic edema (19%, 4/21) was consistent with the literature (15–30%) (3). Areas of restricted diffusion can be reversible or, conversely, progress to frank infarction (58). For instance, Kinno et al. (49) reported a case of PRES with reversible cerebral vasoconstriction syndrome due to ephedrine overdose. Initial brain MRI showed vasogenic edema in the left occipital and both parietal lobes with some hyperintense lesions on DWI with restricted diffusion on ADC maps, indicating the co-existence of cytotoxic edema. Follow-up MRI 1 month later showed residual partial hypertense areas in the superior parietal gyri bilaterally (49). Persistence of brain lesions on follow-up imaging (25, 49, 51) found in 12% of patients (3/25) is a proportion classically reported in various series of PRES.

Imaging analysis did not highlight a specific pattern for poisoning-associated PRES but reinforces the fact that PRES is neither only posterior nor always reversible.

### Are Toxic PRES Different According to the Causative Agents?

#### Similarities

Neurological complications such as hemorrhage (16, 20, 30, 31, 36, 43), reversible cerebral vasoconstriction syndrome (16, 49), and infarction (28, 49) occurred independently of toxic etiology, i.e., kratom (20), alcohol (28, 30, 31), drug overdose (36, 49), and licorice (16, 43).

##### Vasoconstriction and endothelial dysfunction

Most of the pharmacological and toxicological agents involved in this review are known to induce either or both vasoconstriction and endothelial dysfunction. High alcohol concentrations have been associated with dose-related vasoconstriction and impaired dilatation of cerebral vessels (22). In addition, chronic alcoholism and long-term lithium exposure increase reactive oxygen species and nitric oxide in brain endothelial cells. This oxidative stress can induce endothelial dysregulation, leading to the BBB breakdown (27, 34). Cocaine, amphetamines, lysergic acid amide, mephedrone, kratom, and phenylpropanolamine have sympathetic and/or serotoninergic effects that cause vasoconstriction or vasculitis, leading to severe hypertension (36, 59).

In alcohol withdrawal, the hypothalamic–pituitary–adrenal system is stimulated, leading to increased levels of catecholamine and AVP, which can induce acute hypertension (27, 28). Similarly, the biologically active component of licorice, glycyrrhizic acid, inhibits 11 β-hydroxysteroid dehydrogenase, leading to hypervolemic hypertension (16). After snakebite envenomation and organophosphate severe poisoning, neurotoxins (14) and nicotinic stimulation (60), respectively, lead to autonomic dysregulation with massive release of catecholamines and angiotensin II. These effects, in combination with the presence of sympathetic denervated vasculature in the brain posterior area, may induce failure of the cerebral autoregulatory system (14). The increase in proinflammatory mediators, either exogenous, originating from the substance (e.g., venom itself), or endogenous (e.g., in acute alcoholic pancreatitis, induced by venom), can damage the BBB and is likely to cause the leakage of blood contents into the surrounding brain tissue (14, 30).

##### Cerebral hypoperfusion

In several reports, angiography showed multiple areas of narrowing of the intracranial arteries (16, 28, 36, 37, 49), especially in drug overdose (36, 37, 49) with sympathomimetic agents such as ephedrine (49) and phenylpropanolamine (36). Interestingly, in ephedrine overdose–induced PRES (49), although MRI revealed bilateral superior parietal lesions, single-photon emission computed tomography showed selective hypoperfusion in the left superior parietal region.

##### AVP axis hyperstimulation

A recent review highlighted that AVP overstimulation seems to be involved in PRES development and subsequent symptoms, in particular because of both its pathophysiological mechanism in brain edema formation and its involvement in most PRES etiologies (5). AVP hypersecretion could be the trigger of PRES through a dysregulation of ionic/water transglial flux via astrocytic ion channel dysfunction and subsequent brain edema. In the periphery, AVP receptor stimulation could be responsible for symptoms usually reported in PRES such as hyponatremia, acute hypertension, and impaired renal function (5, 61). The use of cocaine, amphetamine (62), and lysergic acid diethylamide (63) and co-administration of alcohol with disulfiram (5) are known situations to stimulate AVP neurons. Thus, these agents could be directly responsible for the pharmacological cascade described above. In several cases, patients received multiple psychotropic drugs such as quetiapine (20, 33, 34), duloxetine (33), sertraline (33), amitriptyline (33), escitalopram (51), and valproate (51); it could be argued that these drugs, known to induce AVP release (64) and cerebrovascular effects, may serve as a contributing factor in the genesis of PRES.

In hepatic encephalopathy, in addition to dysregulation of cerebral blood flow and consequent cerebral vasodilation induced by hyperammonemia (32), ammonia reaching the astrocytes is detoxified in glutamine, and its overproduction promotes, through AVP stimulation, astrocytic swelling, resulting in brain edema (65). In contrast, acute alcohol intoxication (62) and licorice (66) inhibit AVP release, and lithium inhibits renal effects of AVP (5). In PRES associated with alcohol withdrawal (26–28), licorice (44), and lithium intoxication (33, 34), the onset of neurological symptoms ranged from 2 to 9 days after intoxication; this chronology of events may suggest a rebound phenomenon on the AVP axis (5).

#### Specific Characteristics

Visual hallucinations have only been reported in concomitant administration of disulfiram with alcohol (22) and in alcohol withdrawal (26, 27). Visual hallucinations may be a symptom of delirium tremens but also occur in patients with PRES (2). In natural toxin–and chemical substance–associated PRES, seizure was the most frequent symptom, accounting for 67% (10/15) of cases (12–14, 16, 17, 38, 45–48). Similarly, alertness disorders were at the forefront (92%, 11/12) in alcohol-associated PRES (22–32).

##### Multiple risk factors in substance abuse patients

In substance abuse–related PRES, patients had several risk factors for PRES such as chronic hypertension (18, 19), chronic kidney disease (18, 19), and HIV infection (18). In these cases, the different risk factors should act synergistically on the different pathophysiological pathways leading to PRES. For instance, cocaine has the ability to synergistically increase the pathologic processes induced by HIV infection (18) including endothelial dysfunction and disruption of the BBB integrity. Interestingly, single nucleotide polymorphisms in AVP gene and AVP V1a receptor have been associated with drug abuse disorders (67), suggesting a different probability of PRES occurrence in patients with substance abuse.

In patients with multiple risk factors of PRES or in the context of multiple drug intake (20), causality assessment is difficult. In addition, combined drug intoxication also exposes to a risk of drug cocktail effect. As with drug–drug interactions, illicit drug–drug interactions can occur both at the pharmacodynamic (i.e., interactions in which drugs influence each other's effects directly) and pharmacokinetic level (i.e., modification of drug absorption, distribution, metabolism, or excretion). In pharmacodynamic illicit drug–drug interactions, the neurotoxicity and/or endothelial toxicity of both the drug and illicit drug act synergistically, which can promote the occurrence of PRES. Legriel et al. (21) reported a case of lysergic acid amide–induced PRES in a depressed patient treated with clomipramine. The analysis of urinary catecholamines and serotonin metabolites showed a massive sympathetic storm with high urinary serotonin levels, supporting the pharmacodynamic convergence of these two adrenergic and serotoninergic agents. Castillo et al. (20) reported a case of kratom-induced PRES in a patient on fluoxetine for depression with dextroamphetamine misuse. Kratom exerts α2-adrenoreceptor agonistic effects and is known to induce high blood pressure, particularly when combined with other drugs (68). Mitragynine, the major indole-based alkaloid of kratom, is extensively metabolized by hepatic cytochrome P450 (CYP) isoform 3A4 and 2D6 (69). Amphetamine metabolism primarily involves CYP2D6 (70). Fluoxetine and its main metabolite, norfluoxetine, are well-known inhibitors of CYP2D6 and CYP3A4, respectively (71). Taken together, a probable pharmacokinetic illicit drug–drug interaction occurred between mitragynine/dextroamphetamine and fluoxetine, causing overexposure in mitragynine and dextroamphetamine. This case of kratom/amphetamine interaction shares the pharmacological characteristics of PRES induced by clonidine, another α2-adrenoreceptor agonist, and dextroamphetamine overdose described by Minhaj et al. (35).

##### Coagulopathy

Characteristics of PRES associated with snake bite included normal blood pressure (38, 39, 42), coagulopathy (12, 38, 39), and respiratory failure (12, 38). The mechanism of PRES associated with snake venom does not appear to be related to direct toxic effects of the venom within the central nervous system, because venom proteins do not cross the BBB. Instead, neurological manifestations are most often related to blockage of the neuromuscular transmission, causing paralysis, and abnormalities in the coagulation cascade, producing cerebrovascular events (38).

### A Lack of Standardization in Reported Data on Poisoning-Induced PRES

The assessment of the methodological quality of the cases showed that the data reported are very heterogeneous, with a median overall score of 5/10 (IQR 3–6). In addition, none of the case reports met the domain selection criteria (Supplementary Table 1). An unclear selection approach leaves the reader with uncertainty as to whether this is the whole experience of the researchers or only the most serious case, and suggests possible selection bias. As the scientific literature on PRES is almost exclusively represented by case series and case reports, we propose a checklist with various items that should at least be reported in case reports of substance-induced PRES (Supplementary Table 4). Indeed, it seems essential to standardize the reporting of outcome so that studies collecting a large amount of data can be conducted.

## Conclusions

PRES in a context of poisoning does not exhibit a specific imaging pattern. The predominance of various anatomical implications outside the parieto-occipital lobes in PRES, including toxic etiologies, is a key message for clinicians and radiologists. The prevalence of the neurological symptoms was also consistent with the published literature on other etiologies of PRES. In addition to toxic exposure, one-third of patients had at least one other risk factor of PRES. Chronic hypertension (17%) was less frequent than reported in other causes of PRES.

As in iatrogenic PRES, toxicology cases do not always have a temporally close relationship. PRES occurring after 3 days seems to be more related to a rebound effect of poisoning, to secondary brain damage, or to complications related to the management of the poisoning, whereas a shorter period would support a direct link between the causative agent and PRES. Poisoned patients may have a lower threshold for developing PRES. It may also be caused by the convergence of various pathophysiological processes (e.g., high blood pressure, endothelial dysfunction, AVP axis overstimulation) induced directly by the poison and/or indirectly by acting in concert with other factors such as drug–drug interaction or hypomagnesemia that ultimately causes the cerebrovascular cascade leading to PRES.

Although less described, PRES in a context of poisoning, which shares most of the clinical and radiological characteristics of other etiologies, is not to be ignored. The characterization of the pathophysiological mechanisms is essential to individualize management according to the presence of risk factors and/or specific features of PRES. Standardization of data reported in future case series of substance-induced PRES is required in order to better characterize and thus manage this syndrome.

## Data Availability Statement

Publicly available datasets were analyzed.

## Author Contributions

BL, CS, and SE conceived the idea. BL wrote the manuscript and performed the selection and summary of published literature on PRES in clinical toxicology. DB, CV-V, CS, and SE helped to design, write, and revise the paper. CC contributed in improving the article, notably enriching the analysis of neuroimaging data.

### Conflict of Interest

The authors declare that the research was conducted in the absence of any commercial or financial relationships that could be construed as a potential conflict of interest.
