# Supplementary material for: Posterior Reversible Encephalopathy Syndrome in Clinical Toxicology: A Systematic Review of Published Case Reports
Source: Front Neurol. 2020 Feb 12;10:1420. doi: 10.3389/fneur.2019.01420 (PMC7029435; doi:10.3389/fneur.2019.01420)
Supplement: Supplementary file 1 [file Data_Sheet_1.pdf]

Supplementary Appendix

Figure 1 - Flowchart of the literature search.

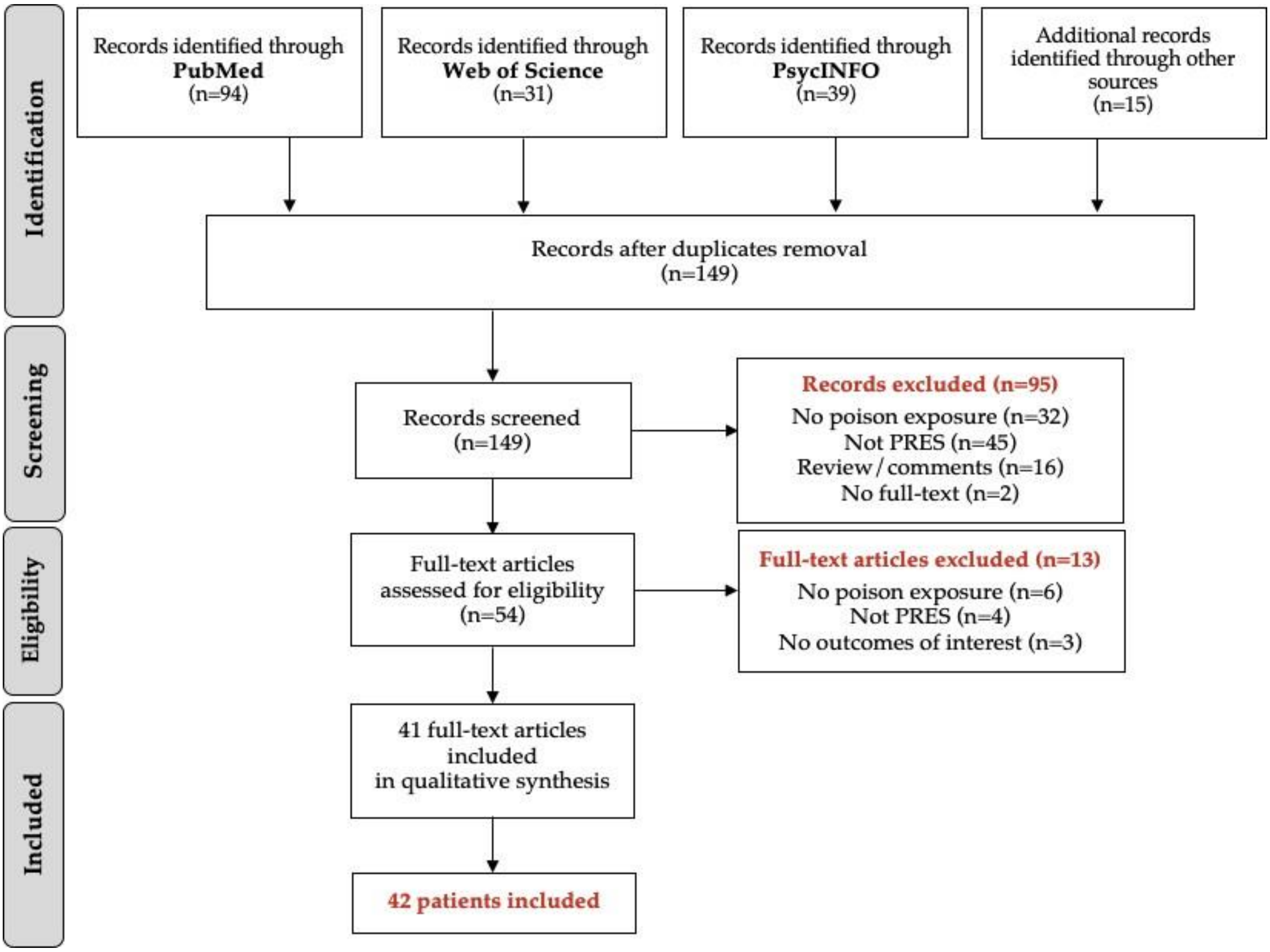

Table 1 ~ Methodological quality assessment of the included cases

Domains	Leading explanatory questions	Points†	Results‡
Selection	1. Does the patient(s) represent(s) the whole experience of the investigator (center) or is the selection method unclear to the extent that other patients with similar presentation may not have been reported?	1	0 (0)
Ascertainment	Was the exposure adequately ascertained?		
	2. Was the time to onset of PRES adequately reported?	1	35 (83.3)
	3. Was the assessment of exposure performed?<br>- Dose and/or urine/serum detection, if applicable.	1	21 (61.8) §
Diagnosis	4. Were imaging features adequately reported?	1	21 (50.0)
	5. Were PRES symptoms and risk factors of PRES (e.g., chronic hypertension, kidney failure) adequately reported?	1	32 (76.2)
Causality	6. Was the dechallenge phenomenon adequately reported?<br>- Time required for symptoms resolution from poison discontinuation and/or additional therapy used (i.e., anticonvulsant, anti-hypertensive agents) have been reported.	1	24 (57.1)
	7. Were other alternative causes that may explain the observation ruled out?	1	18 (42.9)
	8. Were the pharmacological properties of causative agent adequately reported?<br>- Pharmacological properties described in order to potentially explain PRES onset (i.e.,, high-blood pressure and/or endothelial dysfunction).	1	22 (52.4)
Follow-up	9. Was follow-up long enough for outcomes to occur?<br>- Clinical and radiological follow-up performed 3 months later PRES onset.	1	7 (16.7)
Reporting	10. Is the case(s) described with sufficient details to allow other investigators to replicate the research or to allow practitioners make inferences related to their own practice?	1	25 (59.5)
	**Overall score**	**/10**	**5 (3 – 6)**

† Dichotomous items: each "yes" positive response to an item was counted as 1
‡ Results are expressed as number and proportion of case reports with positive response, n (%).
The overall score was calculated as a sum of points obtained at each item, median (interquartile range)
§ Exposure assessment was not applicable in 9 case reports

Table 2 ~ Details of methodological quality assessment of the included cases

Ref.	Substance	Q1	Q2	Q3	Q4	Q5	Q6	Q7	Q8	Q9	Q10	Overall
Bazuaye-Ekwuyasi et al. (18)	Cocaine	0	0	1	0	1	1	1	1	0	1	6
Dasari et al. (19)	Cocaine	0	1	1	1	1	1	1	1	0	1	8
Tantikittichaikul et al. (52)	Amphetamine	0	0	1	0	0	0	0	0	1	0	2
Omer et al. (50)	Mephedrone	0	1	0	0	1	1	0	0	0	0	3
Castillo et al. (20)	Kratom	0	0	1	0	1	0	1	1	0	0	4
Legriel et al. (21)	LSA	0	1	0	1	1	1	1	1	0	1	7
Bhagavati et al. (22)	Acute Alcohol	0	1	1	0	0	0	0	0	0	0	2
Coppens et al. (23)	Acute Alcohol	0	1	1	1	1	1	1	1	0	1	8
Srikrishna et al. (24)	Acute Alcohol	0	1	0	1	1	1	0	0	0	1	5
Kim et al. (25)	Acute Alcohol	0	1	0	1	1	1	1	1	0	1	7
Ishikawa et al. (26)	Alcohol withdrawal	0	1	n/a	1	1	1	1	0	0	1	6
Mengi et al. (27)	Alcohol withdrawal	0	1	n/a	0	1	1	1	1	0	1	6
Gill et al. (28)	Alcohol withdrawal	0	1	n/a	1	1	1	0	0	0	1	5
Kimura et al. (29)	Chronic Alcohol	0	1	n/a	1	1	0	1	1	0	1	6
Baek et al. (30)	Chronic Alcohol	0	1	n/a	1	1	1	0	1	0	1	6
Magno Pereira et al. (31)	Chronic Alcohol	0	1	n/a	1	1	1	0	1	0	1	6
Murphy et al. (41)	Chronic Alcohol	0	0	n/a	0	0	0	0	0	0	0	0
John et al. (32)	Chronic Alcohol	0	1	n/a	0	0	0	0	0	0	0	1
Fitzgerald et al. #1 (33)	Lithium	0	1	1	1	1	0	0	1	1	0	6
Fitzgerald et al. #2 (33)	Lithium	0	1	1	1	1	0	0	1	1	0	6
Loens et al. (34)	Lithium	0	1	1	1	1	1	0	0	0	1	6
Minhaj et al. (35)	Dextroamphetamine	0	1	1	0	1	1	0	0	0	1	5
Mann et al. (51)	Acetaminophen	0	1	1	0	1	1	1	0	0	1	6
Kinno et al. (49)	Ephedrine	0	1	0	1	0	0	1	0	0	0	3
Kawanabe et al. (36)	PPA	0	1	0	1	1	1	1	1	0	1	7
Weidauer et al. #1 (37)	Digitoxin	0	1	1	0	0	0	0	0	0	0	2
Akinci et al. (15)	Bismuth	0	1	1	0	1	1	0	0	0	1	5
Delgado et al. (38)	Snake	0	1	1	0	0	0	0	1	1	0	4
Varalaxmi et al. (42)	Snake	0	1	1	0	0	0	0	0	0	0	2
Ibrahim et al. (39)	Snake	0	0	1	1	1	1	1	1	0	1	7
Kaushik et al. (12)	Snake	0	1	1	0	0	1	0	0	1	1	5
Marrone et al. (13)	Scorpion	0	1	1	0	1	0	0	0	0	0	3
Rebahi et al. (14)	Scorpion	0	1	1	0	1	1	0	1	1	1	7
Loh et al. (40)	Wasp stings	0	0	0	0	1	0	1	0	0	0	2
Du et al. (48)	Wasp stings	0	1	0	1	1	0	0	0	0	1	4
Chatterjee et al. (16)	Licorice	0	1	0	1	1	0	1	1	1	0	6

Van Beers et al. (43)	Licorice	0	1	0	0	1	0	0	1	0	0	3
Morgan et al. (44)	Licorice	0	1	0	0	1	1	0	1	0	1	5
O'Connell et al. (45)	Licorice	0	0	0	1	1	0	1	1	0	1	5
Tassinari et al. (17)	Licorice	0	1	1	1	1	1	1	1	0	1	8
Zhou et al. (46)	Mushroom	0	1	0	0	0	1	0	0	0	0	2
Phatake et al. (47)	Organophosphate	0	1	1	1	1	1	1	1	0	1	8

n/a: not applicable

Table 3 ~ Summary of the clinico-radiological characteristics of the 42 included patients

**Demographic data**		
Age, median (IQR)	41 (27 – 55)	
Female sex, n (%)	22 (52.4)	
**Risk factors for PRES**, n (%)	**14 (33.3)**	
Chronic hypertension	7 (16.7)	(18,19,28,33,34,45)
Chronic kidney disease	2 (4.8)	(18,19)
Acute kidney injury	8 (19.0)	(15,28,33,34,40,42,51)
Acute pancreatitis	3 (7.1)	(26,30,41)
**Substance involved**, n	**19**	
**Alcohol, n (%)**	**12 (28.6)**	(22–32,41)
**Drugs**, n (%)	**9 (21.4)**	
Lithium	3	(33,34)
Dextroamphetamine	1	(35)
Acetaminophen	1	(51)
Ephedrine	1	(49)
Phenylpropanolamine	1	(36)
Digitoxin	1	(37)
Bismuth	1	(15)
**Illicit drugs**, n (%)	**6 (14.3)**	
Cocaine	2	(18,19)
Amphetamine	1	(52)
Mephedrone	1	(50)
Kratom	1	(20)
Lysergic acid amide	1	(21)
**Natural toxins**, n (%)	**14 (33.3)**	
Snake bite	4	(12,38,39,42)
Scorpion sting	2	(13,14)
Wasp sting	2	(40,48)
Licorice	5	(16,17,43–45)
Mushroom	1	(46)
**Chemical substance**, n (%)	**1 (2.4)**	
Organophosphate	1	(47)
**Initial presentation**, n (%)		
Alertness disorders	27 (64.3)	(13–15,18–40,46)
Headache	20 (47.6)	(13,16,17,19–21,24,31–33,35,36,38,42–45,47,48,52)
Visual disturbance	23 (54.8)	(12,13,15,16,19–21,23,25–27,29,34,35,37,39,41–47)
Seizure	21 (50.0)	(12–17,21,24,26,31–34,36,38,45–48,50,51)
Acute high blood pressure	28 (70.0)	(12,14,16–21,23–25,27–29,31,33–36,40,43–47,50–52)
**Imaging findings**, n (%)		
**Anatomical pattern**		
**Atypical variant**	**6 (14.3)**	(15,29,36,43,50,52)
Occipital	4	(29,36,43,50)
Cerebellum	2	(15,52)
**Parieto-occipital pattern**	**8 (19.0)**	(12,16,23,27,37,40,42,45)
**Combined pattern**	**28 (66.7)**	(13,14,17–22,24–26,28,30–35,38,39,41,44,46–49,51)
Parieto-occipital	23	(13,14,17,18,20–22,24–26,28,30,33,38,39,41,44,46–49,51)
Occipital	1	(34)
Parietal	3	(19,31,32)
Frontal	17	(13,17,18,20,22,24–26,28,31,33,38,44,46,48,49,51)
Temporal	7	(18,25,26,30,32,33,47)
Cerebellum	8	(14,18–21,30,35,41)
Midbrain and/or brainstem	4	(18,19,21,35)
Basal ganglia and/or thalami	5	(18,19,21,34,46)

**Lesion characteristics**		
Symmetrical patterns	25/33 (75.8)	(12–15,19,22–25,27,29,32–34,36,37,39,40,43,44,46,47,49,50)
Positive DWI	12/20 (60.0)	(20,25,26,29,30,36,37,39,47–49)
Arterial constriction	5/6 (83.3)	(16,28,36,37,49)
**Nature of edema**		
Vasogenic	18/21 (85.7)	(16,17,19,21,23,25,26,28–31,33,34,36,45,47–49)
Cytotoxic	4/21 (19.0)	(20,24,39,49)
**Lesion reversibility**	**22/25 (88.0)**	(12–14,16,18,21,22,24,26–31,33,38,43–47,50)
**Neurological complications**		
Hemorrhage	6 (14.3)	(16,20,30,31,36,43)
Infarction	2 (4.8)	(28,49)

Table 4 ~ Proposed checklist items to improve substance-induced PRES reporting

Items	Comments
**Selection**	Please precise if the patient(s) represent(s) the whole experience of the investigator (center)
**Comorbidities**	
Demographic data	
History of chronic hypertension	
Chronic kidney disease	
History of substance abuse	
Chronic treatments	To assess drug causality and drug-drug interactions in the occurrence of PRES
Other relevant comorbidities	e.g., cancer, sepsis, autoimmune disorders, transplantation
**Exposure**	
Substance	
Dose/estimated ingested dose	
Drug detection	If applicable
Time to onset	Time to onset from start/stop exposure and neurological disturbances and brain imaging
**Presentation**	Please specify the time course
**Clinical**	
Blood pressure	
Symptoms	
**Serum biochemical**	
Creatinine	
Sodium	
Calcium	
Magnesium	
Lactate dehydrogenase	
Albumin	
**CSF biochemical**	If applicable
Protein	
**Radiological diagnosis**	
Sequences and location	
Brain imaging pattern	i.e., dominant parieto-occipital, holohemispheric watershed, superior frontal sulcal or another pattern
Diffusion-weighted imaging	
Apparent diffusion coefficient	
**Differential diagnosis**	Please specify ruled out diagnosis
**Symptomatic treatments**	Please specify the timeline
Drugs administration	
Drugs discontinuation (dechallenge)	
Rechallenge	Please specify if rechallenge was performed
Symptoms resolution	
Biochemical resolution	
**Hospital discharge (day)**	
**Follow-up**	Relevant follow-up with distance needed
Clinical resolution	
Radiological resolution	
